# Nanobubbles in vase water inhibit transpiration and prolong the vase life of cut chrysanthemum flowers

**DOI:** 10.1002/pei3.10124

**Published:** 2023-10-24

**Authors:** Rie Nakazawa, Akito Tanaka, Naoki Hata, Hisato Minagawa, Emiko Harada

**Affiliations:** ^1^ School of Environmental Science The University of Shiga Prefecture Hikone Shiga Japan; ^2^ Graduate School of Engineering The University of Shiga Prefecture Hikone Shiga Japan; ^3^ School of Engineering The University of Shiga Prefecture Hikone Shiga Japan

**Keywords:** chrysanthemum, cut flower, nanobubble water, transpiration, water absorption

## Abstract

Nanobubble (NB) water has been shown to promote the growth of several types of plants and animals, but the mechanism underlying this promoting effect remains unclear. The present study evaluated the mechanism by which NBs maintain the freshness of cut flowers by keeping cut chrysanthemum (*Chrysanthemum morifolium* Ramat.) flowers at the bud stage in vase water containing air NBs. The condition of petals and leaves was assessed to determine the vase life of these cut flowers. The NB treatment delayed bud opening and petal senescence of the inflorescences. Water absorption and transpiration by cut flower stems were lower in NB water than in distilled water (DW). Furthermore, when all the leaves were removed from the cut flower stems, no significant difference in vase life was observed between NB water and DW. These findings indicate that the inhibition of transpiration from leaves prolonged the vase life of NB‐treated cut chrysanthemum flowers. In the early stage of the treatment, NB treatment significantly reduced transpiration without closing stomata, suggesting that the reduction in transpiration observed in the NB‐treated plants might be due to the suppression of cuticular transpiration, defined as water loss through the epidermis. Surface tension, one of the important driving forces of water movement in plants, was not affected by the presence of NBs in water. To our knowledge, this is the first report to show that transpiration from leaves is inhibited by NB treatment.

## Graphical abstract

Nanobubble (NB) water prolonged the vase life of cut chrysanthemum flowers and reduced the water absorption and transpiration rates of plants. NB water would be an equally effective, but safer, alternative to several types of chemicals currently used to prolong the vase life of cut flowers.

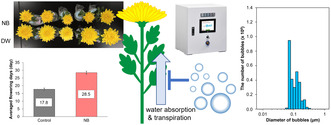

## 1 INTRODUCTION

Nano/ultrafine bubbles (NBs) are fine bubbles less than 1 μm in diameter (Yokoyama et al., 2021). NBs exhibit several unique physicochemical features in water. For example, air and oxygen NBs can maintain high concentrations of dissolved oxygen (Ebina et al., 2013) and generate OH radicals (Ahmed et al., 2018). NBs are stable over several months (Nirmalkar et al., 2018), with this stability thought to result from microscopic particles attached to the bubbles (Ueda et al., 2022). NBs have large specific surface areas, excellent dispensability, and autolytic activity (Matsuki et al., 2012). Therefore, NBs have been used in water treatment, and water containing NBs has been used in biomedical engineering and the synthesis of nanomaterials (Agarwal et al., 2011).

NB water was found to activate the metabolism of plants and animals, indicating that NB water might have agricultural applications (Ebina et al., 2013). For example, water containing NBs was found to improve the seed germination rates of barley (Liu et al., 2013) and tomato (Yokoyama et al., 2021). Furthermore, NB water enhanced the growth of several species of plants, including *Brassica campestris* (Ebina et al., 2013), carrot, fava bean, tomato (Ahmed et al., 2018), and rice (Wang et al., 2021). To date, however, the mechanisms underlying the relationships between the physicochemical properties of NBs and their effects on living organisms have not been determined.

Beneficial methods to extend the vase life of cut flowers are being actively investigated from a variety of perspectives. Factors such as suitable handling, ecological compatibility for a safe environment, non‐harmful properties, and low price are necessary because poor postharvest practices enhance the loss of quality of cut flowers (Nguyen & Lim, 2021). NBs have also been reported to maintain the freshness of cut flowers, including chrysanthemum, rose, trumpet daffodil, tulip (Nakamoto & Nomura, 2013), carnation (Li et al., 2021), and *Cymbidium* Sweetheart (Chung et al., 2022). These results suggest that NBs could prolong the vase life of cut flowers without chemical substances.

Because the freshness of cut flowers is spoiled by over‐transpiration (Kitamura & Ueno, 2015), the present study focused on whether water absorption and movement are related to the vase life of cut flowers treated with NBs. Because the cohesion–tension theory of water transport in plant xylem has indicated the importance of a continuous water column and the mechanism of long‐distance ascent of sap in plants (Domec, 2011), the characteristics of the surface tension of NB water were also examined to determine whether the surface tension of NB water affects the process of water absorption by cut flowers.

## 2 MATERIALS AND METHODS

### 2.1 Generation of NB water

**FIGURE 1:**
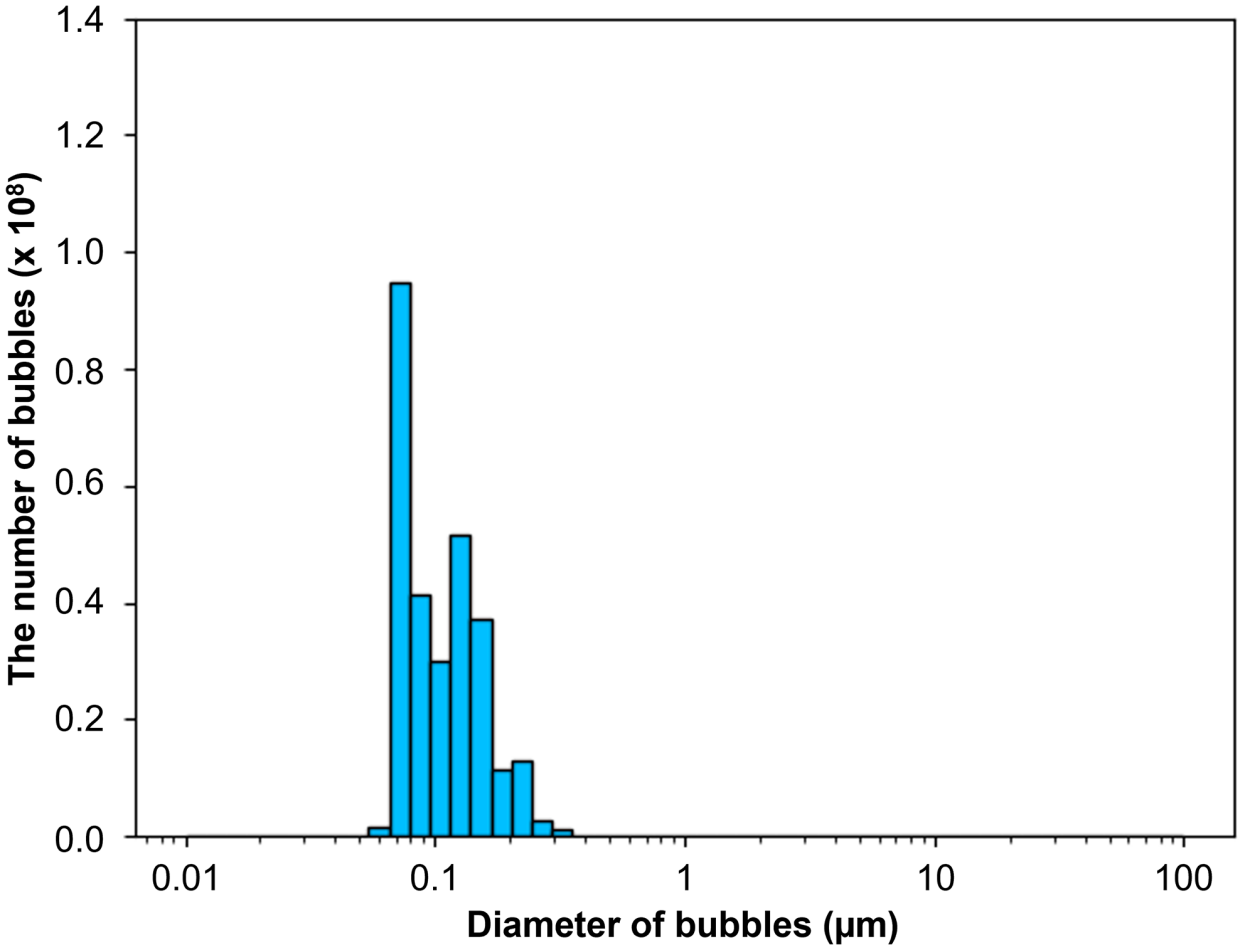
Distribution of the diameters of NBs generated by agriGaLF15 FZ1C‐G15. A total of 2.0 × 10^8^ bubbles were evaluated, with minimum and maximum detectable diameters of 0.056 and 20 μm, respectively. Of these bubbles, 99.98% had diameters less than 1 μm, indicating that almost all of these bubbles could be defined as NBs. NB, nanobubble.

NB water was generated from distilled water (DW) by employing air‐NBs for 10 min at an aeration pressure of 108–112 kPa with agriGaLF15 FZ1C‐G15 (IDEC Corporation). NB water was prepared every 10 days and maintained at room temperature. The size and density of NBs were measured by IDEC Corporation using a SALD‐7500H nano‐particle size analyzer (Shimadzu) based on laser diffraction scattering method. Of the counted bubbles, 99.98% consisted of NBs, with diameters ranging from 0.056 to 0.887 μm and a mode of 0.089 μm (Figure 1).

### 2.2 Plant materials and growth condition

Commercially‐sold cut chrysanthemum (*Chrysanthemum morifolium* Ramat.) plants bearing one visible terminal flower bud each were purchased from a retail shop. The stems of the plants were adjusted to 40 cm in length, and the lower leaves were removed, with 20 upper leaves remaining.

The stem ends of these cut flowers were dipped in glass vessels measuring 5.8 cm (top) × 9.6 cm (body) × 18.1 cm (height) and containing 400 mL of NB water or DW at a depth of 7.5 cm. NB water and DW were added daily to maintain a constant volume, and evaporation from the water surface was minimized by using a styrene board (Styrofoam; DuPont Styro) and plastic wrap (Saranwrap; Asahi Kasei Home Products Corporation). The vases were placed in a growth chamber (BiOTRON LH‐410SP; Nippon Medical & Chemical Instruments) maintained at 25°C, with 11.5‐h light/12.5‐h dark cycles.

### 2.3 Vase life and freshness of cut flowers (Experiment 1)

The freshness of cut flowers (five replicates each in NB water and DW) was evaluated daily for 31 days according to the guidelines recommended by the Japan Flower Promotion Center Foundation (https://www.jfpc.or.jp/doc/himochi/ver2020/28.pdf). Briefly, the opening of the buds, the freshness and senescence (browning) of the inflorescences, and the freshness of the leaves were scored to determine the lifetimes of the cut flowers (Table S1), as described by the above guidelines. Greenness in three leaves per plant was measured daily with the Soil and Plant Analysis Development (SPAD) chlorophyll meter (SPAD‐520Plus; Konica Minolta) for 31 days, with the relative amount of chlorophyll determined by measuring leaf absorbances in the red and near‐infrared regions.

### 2.4 Water absorption and transpiration of cut flowers (Experiment 2)

To determine water absorption, the water loss from each glass vessel containing cut flower stems (three replicates each in NB water and DW) were recorded daily for 27 days. At the same time, the weights of the plants were also measured to calculate the amount of transpiration. The amount of transpiration (*T*_a_ [g]: weight of transpired water) was calculated as:

(1) $$T_{a}={\mathrm{WA}}_{a}-\left(m_{\text{date−1}}-m_{\text{date}}\right)\;\left[g\right]$$

(2) $${\mathrm{WA}}_{a}={\mathrm{WA}}_{a}^{*}\times D\;\left[g\right]$$

(3) $${\mathrm{WA}}_{a}^{*}=400-{\text{remained water}}_{\text{date}}\;\left[\mathrm{mL}\right]$$

where ${\mathrm{WA}}_{a}^{*}$ [mL] and ${\mathrm{WA}}_{a}$ [g] are the volume and mass, respectively, of water absorbed by each flower, determined by subtracting the water [mL] remaining in each vase at every measurement from the regulated water volume (400 mL) and calculated using formulas (2) and (3), respectively; and *m*_date_ and *m*_date−1_ are the weights of plants on the day of measurement and the previous day, respectively, and *D* = 1.0 g mL^−1^ is the density of water at 293 K.

The transpiration rate of cut flowers (three replicates each in NB and DW) was monitored on days 4–12 using LI‐6400XT series (Meiwafosis), with 10 measurements of the two largest leaves of each individual plant taken every 5 s. Because the leaves of these chrysanthemums did not cover the entire 2 cm × 3 cm chamber opening, the leaf areas inside the chamber were estimated by ImageJ software (https://imagej.nih.gov/ij/index.html) and utilized to calculate transpiration rates.

### 2.5 Evaluation of the effect of defoliation (Experiment 3)

All the leaves of cut flowers (five replicates each in NB water and DW) were removed with scissors. The freshness of each plant and the amount of water absorption were evaluated daily for 20 days as described in Experiments 1 and 2, respectively.

### 2.6 Evaluation of stomatal opening (Experiment 4)

Leaves of chrysanthemum plants (three replicates each in NB water and DW) grown for 3 and 20 days were excised during a light period and immediately placed onto an aluminum stage with sticky carbon tape and frozen in liquid nitrogen. The abaxial sides of the leaves were imaged by scanning electron microscopy (TM‐3030; Hitachi High‐Tech Corporation) and processed using ImageJ software to determine stomatal apertures, pore sizes, and the density of stomata. Stomatal apertures were calculated by dividing the width (μm) by the length (μm) of the stomata (Simeoni et al., 2022). Stomatal pore areas (*S*, μm^2^) were an approximate match ellipse and calculated using the equation:

(4) $$S=\pi \times \left(\frac{\text{width}}{2}\right)\times \left(\frac{\text{length}}{2}\right)\;\left[{\mathrm{\mu m}}^{2}\right]$$

### 2.7 Measurement of surface tension of NB water (Experiment 5)

Water surface tension was measured by the arranged pendant drop method (Kurimoto et al., 2020). Briefly, NB water or DW was poured into a plastic box, and the sizes of the air bubbles rising from the tips of the stainless‐steel tubes were measured after the generation of NB to determine the surface tension of water. The measurements were repeated 10 times.

### 2.8 Statistical analysis

Time‐dependent changes of SPAD values, amounts of water absorption and transpiration, transpiration rates, and leaf stomatal opening were statistically analyzed by linear mixed models (LMMs). These values were considered dependent variables and the treatment (NB water or DW), day of measurement, and their interaction considered independent variables. When the effect of treatment was statistically significant (*p* < .05), differences between NB water and DW were assessed on each day of measurement, and days with significant differences identified. Benjamini–Hochberg method was performed to control the false discovery rate. All analyses were performed using R 4.3.1 software (https://www.r‐project.org/), with *p* < .05 defined as statistically significant.

## 3 RESULTS

### 3.1 Vase life and freshness of cut flowers (Experiment 1)

**FIGURE 2:**
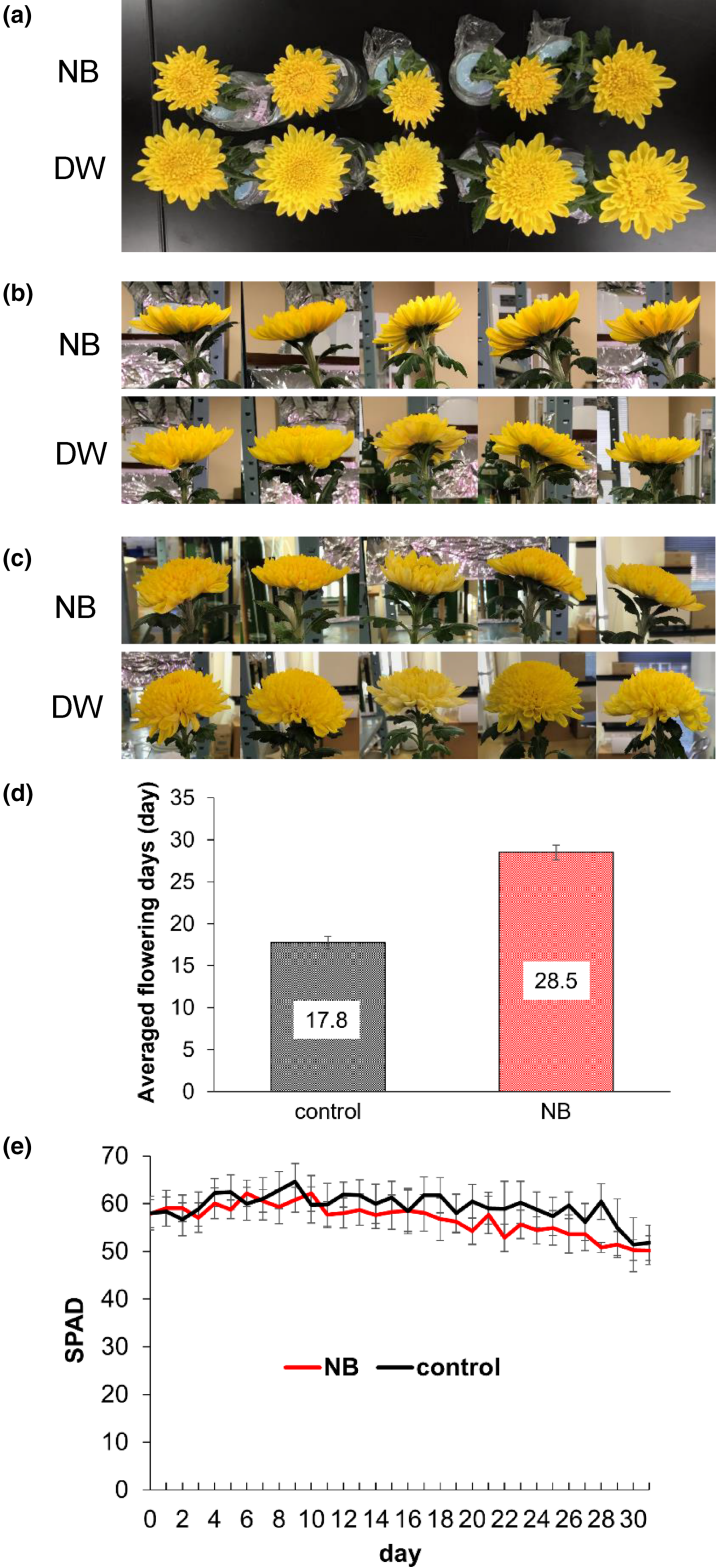
Cut chrysanthemum flowers treated with NB water or DW. (a–c) Inflorescences after treatment with NB water or DW (control) for (a, b) 10 days and (c) 21 days. The openings were more prominent in DW than in NB water, with inflorescences in NB water remaining fresh on day 21. (d) Vase life of cut flowers treated with NB water or DW was scored based on the guidelines for freshness of cut chrysanthemum flowers as shown in Table S1. Bars indicate SE (*n* = 5 each). (e) Chlorophyll contents of the leaves in NB water and DW. Bars indicate SE (*n* = 5 each). Statistically significant differences were not detected by linear mixed model during the experiment. DW, distilled water; NB, nanobubble; SPAD, soil and plant analysis development.

The cut flowers treated with NB water had a longer life (28.5 ± 1.4 days) than those treated with DW (17.8 ± 1.3 days), with the bud opening being delayed in NB water compared with DW (Figure 2a–d). Chlorophyll contents evaluated by SPAD measurements, however, did not differ significantly in the leaves of cut flowers treated with NB water and DW (Figure 2e).

### 3.2 Water absorption and transpiration of cut flowers (Experiment 2)

**FIGURE 3:**
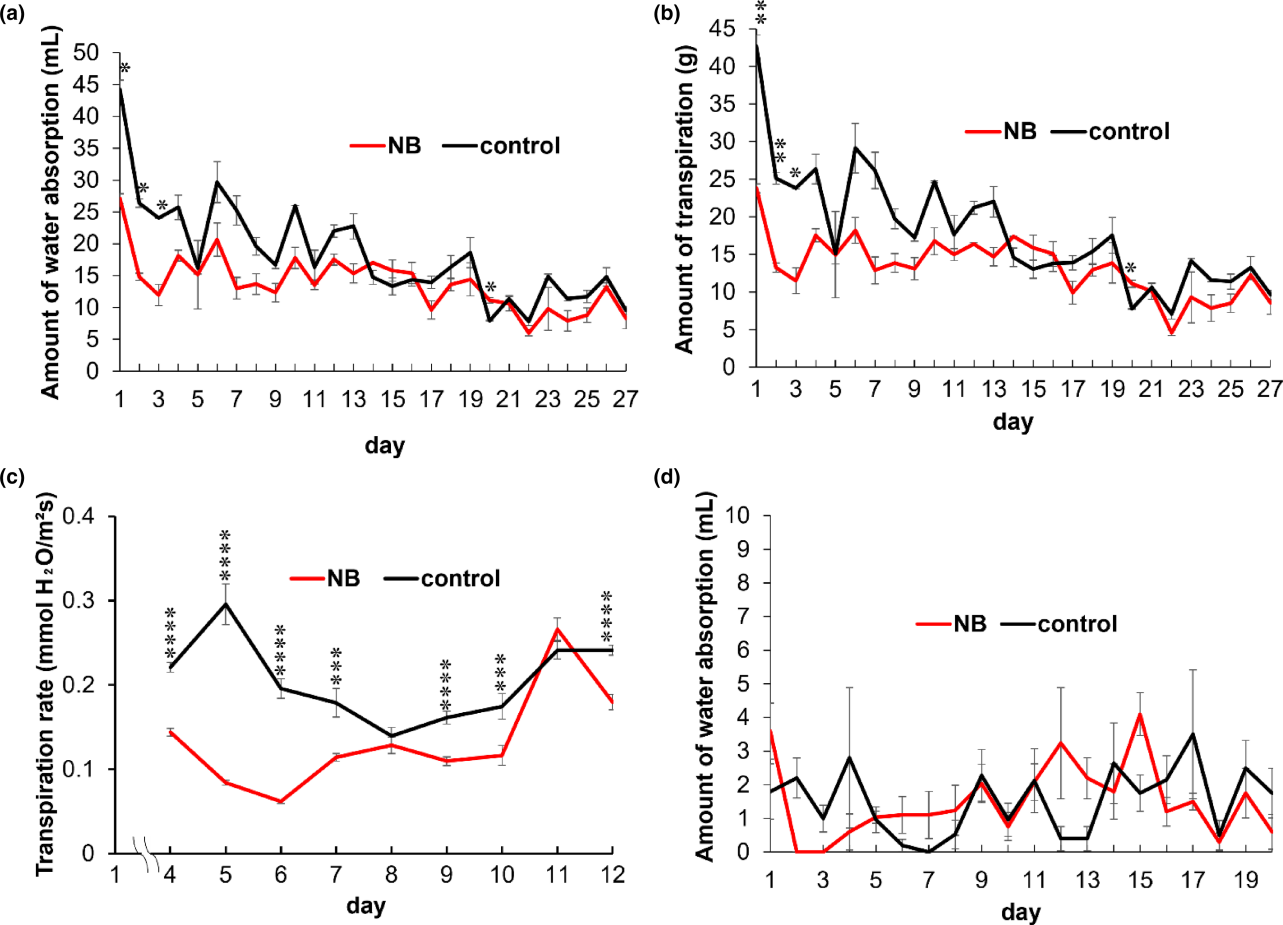
Vase life and water consumption of cut chrysanthemum flowers. (a) Water absorption and (b) amount of transpiration in NB water and DW (control). Bars indicate SE (*n* = 3 each). (c) Transpiration rate (mmol H_2_O m^−2^ s^−1^) over time in cut flowers treated with NB water and DW. Bars indicate SE (*n* = 60 each). (d) Amount of water absorption after removal of all leaves. Bars indicate SE (*n* = 5 each). *p < 0.05; **p < .01; ***p < .001; ****p < .0001 compared with DW (control). DW, distilled water; NB, nanobubble.

Over the experimental period, water absorption (WA_a_) was significantly lower in cut flower stems treated with NB water than with DW (*p* = .002, Figure 3a; *p* = .004, Figure S1). Furthermore, the amount of transpiration (*T*_a_; *p* = .001, Figure 3b) and the transpiration rate (*p* = .039, Figure 3c) were significantly lower in NB water than in DW. The differences in both WA_a_ and *T*_a_ between NB water and DW were greater during the first half than during the second half of the experimental period (Figure 3a,b). The transpiration rate of plants was lower in NB water than in DW on most days during the experimental period (Figure 3c).

### 3.3 Evaluation of the effect of defoliation (Experiment 3)

Removal of all the leaves of cut flowers markedly reduced absorption of both NB water and DW (Figure 3d). The mean amounts of NB water and DW absorbed per day by cut flowers were 1.51 and 1.52 mL, respectively, with LMM analysis showing no significant difference between NB water and DW. Furthermore, the mean vase life of defoliated plants was similar in flowers treated with NB water (11.8 days) and DW (12.0 days); however, vase life was shorter than that of non‐defoliated plants treated with NB water (28.5 days) and DW (17.8 days).

### 3.4 Evaluation of stomatal opening (Experiment 4)

**FIGURE 4:**
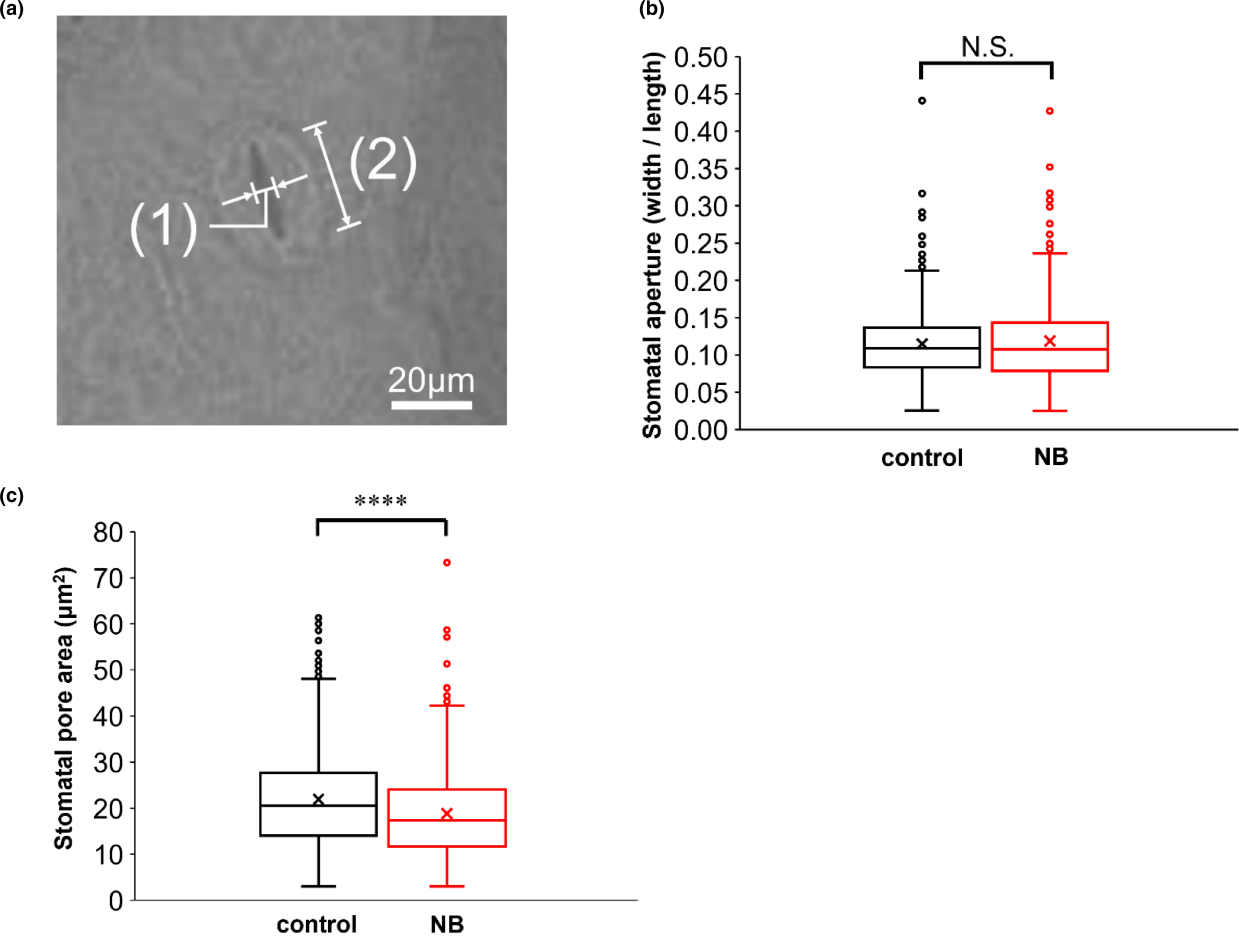
Stomatal apertures of leaves of cut chrysanthemum flowers in NB water and DW. (a) Widths (1) and lengths (2) of stomata, as estimated by scanning electron microscopy. (b) Openings of stomata (width divided by length) in leaves of cut flowers maintained for 20 days in NB water (*n* = 467) and DW (control, *n* = 535). The horizontal line in each box indicates the median; the lower and upper lines indicate the 25th and 75th percentiles, respectively; the short lines on the top and bottom of each box indicate the maximum and minimum numbers, respectively, and the cross mark indicates the average. The points removed from the boxes indicate outliers. N.S., not significant by linear mixed model. (c) Stomatal pore areas, determined as approximate ellipses, in NB water (*n* = 467) and DW (*n* = 535). ****p < .0001 compared with DW (control). DW, distilled water; NB, nanobubble.

The density of stomata on leaves was similar in cut flower stems treated with NB water and those treated with DW (data not shown). Leaf stomatal characteristics, such as stomatal aperture and pore area, did not differ significantly between cut flower stems in the NB treatment and the DW control on day 3 after the start of treatment (stomatal aperture: *p* = .552, Figure S2a; stomatal pore area: *p* = .675, Figure S2b). On leaves of cut flower stems treated with NB water and DW, the average stomatal apertures were 0.110 ± 0.002 and 0.113 ± 0.003, respectively, and the average of stomatal pore areas were 19.76 ± 0.44 and 20.38 ± 0.52 μm^2^, respectively, on day 3 of the experiment. On day 20 of experiment, the average stomatal apertures on leaves of cut flower stems treated with NB water and DW were 0.119 ± 0.003 and 0.115 ± 0.002, respectively (*p* = .339, Figure 4a,b). The average stomatal pore areas on leaves of cut flower stems treated with NB water were 18.81 ± 0.45 μm^2^, 14% lower than that of stomata on leaves of cut flower stems treated with DW (21.93 ± 0.45 μm^2^) (*p* = 5.786 × 10^−6^, Figure 4c).

### 3.5 Measurement of surface tension of NB water (Experiment 5)

The surface tension of DW was 74.5 ± 0.3 mN m^−1^. This surface tension was not altered by NB treatment for 10 min at an aeration pressure of 108–112 kPa, resulting in a surface tension of 74.8 ± 0.4 mN m^−1^.

## 4 DISCUSSION

### 4.1 Inhibition of transpiration by NBs prolonged vase life of cut flowers

The vase life of cut flowers has been found to be strongly affected by water balance, a parameter determined by a combination of transpiration and water absorption (Ichimura, 2010). The present study showed that NB treatment prolonged the vase life of cut chrysanthemum flowers without changing the chlorophyll contents of the leaves, an indicator of leaf freshness. NB water also significantly reduced water absorption and transpiration, especially during the early stage of treatment. These findings indicate that first‐stage transpiration is associated with NB maintenance of the freshness of flowers, independent of the freshness of leaves.

Inhibition of transpiration has been shown to contribute to extended vase life of cut flowers through the maintenance of turgor (Kitamura & Ueno, 2015). For example, maintaining cut roses in aqueous abscisic acid solution delayed the fading of these flowers (Halevy et al., 1974), and covering the leaves of cut hydrangea flowers with polyvinyl chloride film or removing these leaves extended the vase life of these flowers (Kitamura & Ueno, 2015). The present study showed that defoliation eliminated the difference in water absorption of cut flowers maintained in NB water and DW, indicating that inhibition of transpiration by NB water prolonged the freshness of cut chrysanthemum flowers.

Chemical pulsing treatments have been shown to prolong the vase life of cut chrysanthemum flowers. Chemicals shown to prolong vase life include silver thiosulfate (Ag_2_S_2_O_3_; STS), hydroquinone (HQ), 8‐hydroxyquinoline sulfate (8‐HQS), silver nitrate (AgNO_3_), aminooxyacetic acid (AOA), calcium dichloride (CaCl_2_), cobalt dichloride (CoCl_2_), aluminum sulfate (Al_2_(SO_4_)_3_), chlorine dioxide (ClO_2_), and benzyladenine (BA). More recently, lanthanum (La) and selenium (Se) were shown to prolong vase life (Carillo et al., 2022). Many of the substances used to maintain cut flowers are fungicides or regulator of plant hormones, chemicals that may affect the environment. If NB treatment is found to be as effective as these chemicals, NB water would be a safe alternative for the postharvest treatment of cut flowers.

### 4.2 Cuticular transpiration may be related to vase life of NB‐treated cut flowers

Within the first 3 days of the experiment, NB water significantly reduced water absorption and transpiration without changing the characteristics of leaf stomata. Furthermore, transpiration decreased over time in the cut flower stems treated with DW, although the stomatal pore area was not affected. In addition, during the late stages of the experiment, the stomatal pore area was reduced by 14% in the NB treatment, but transpiration did not differ significantly between the NB treatment and the DW control. These results suggest that there is no direct relationship between stomatal opening and transpiration rate and support our hypothesis that the reduction in transpiration in the NB treatment may have been due to the suppression of cuticular transpiration, that is, reduced water loss across the cuticular layer (Hasanuzzaman et al., 2017). The diameters of the NBs suggest that these NBs may be stuck on the pores of cuticles in the leaf interior, obstructing the passage of water. For example, permeability experiments using the leaves of *Hedera helix* showed that the sizes of passable hydrophilic pathways in cuticular membranes were normally distributed, with a mean ± standard deviation pore radius of 0.30 ± 0.02 nm (Popp et al., 2005). Depending on the species and conditions, water loss through the cuticles can be as high as 28% of the water transpired through the stomata (Hasanuzzaman et al., 2017). This hypothesis was supported by the high cuticular transpiration rates observed in chrysanthemum plants, which were likely due to low epicuticular wax development (Rajapakse et al., 1988).

Surfactant‐coated NBs have been observed in the xylem sap of several temperate angiosperms, suggesting that these NBs may have originated spontaneously from multiphase interactions at the pit membranes (Guan et al., 2022). Integrating the actions of “artificial” and “natural” NBs in conduit fluid may lead to a better understanding of water movement within plants.

### 4.3 Effect of surface tension of NB water on water movement in cut flowers

Within a plant, the interface between liquid water and air lies at the surface of cell walls, with surface tension being an important driver of water movement in plants (Fisher, 2000). The addition of a surfactant, such as polyoxyethylene lauryl ether, to vase water reduces its surface tension and increases the matric potential of water sucked into the xylem vessels, resulting in rapid hydration in cut chrysanthemum flowers (Doi & Tsuruga, 2009). The ability of NB treatment to increase the surface tension of water (Park et al., 2001) may result in a reduction of transpiration by cut flowers through a reduction in hydration. The present study, however, found that NB treatment did not affect the surface tension of water, in good agreement with previous findings (Matsumoto & Tanaka, 2008). The results of the present study indicate that the surface tension of NB water is not related to the reduction of water movement. In contrast, another study reported that the surface tension of NB water, which ranged from 40 to 60 mN m^−1^, was lower than that of ion‐exchanged water (Amaki, 2017). These contradictory results may be due to the effects of impurities in NB water on surface tension (Matsumoto & Tanaka, 2008; Yasui et al., 2019). Additional studies are required to determine whether NB treatment affects the transpiration of cut flowers by altering the surface tension of water.

## 5 CONCLUSION

NB water is expected to have a variety of industrial and agricultural applications, but its physical properties and effects on living organisms remain largely unknown. NBs have also been reported to maintain the freshness of cut flowers of several plant species. Although several chemicals are currently utilized to prolong the vase life of cut chrysanthemum flowers, but NB water would be a safer alternative. The present study found that water absorption and transpiration of cut chrysanthemum flowers were lower in NB water than in DW, indicating that the inhibition of transpiration by NB water contributed to its extension of the vase life of flowers. NB treatment, however, did not alter the characteristics of leaf stomata in the early stage, which are closely related to transpiration, suggesting that the NB‐associated reduction in transpiration might be due to the suppression of cuticular transpiration or water loss through the epidermis. Surface tension was not affected by the presence of NBs in water, making the mechanism by which the physicochemical features of NB water affect plants unclear. Additional studies are needed to determine the detailed mechanisms by which NBs affect the vase life of cut flowers and to promote the practical applications of NBs.

## FUNDING INFORMATION

This work was supported by JSPS KAKENHI (Grant Number 20K06324).

## CONFLICT OF INTEREST STATEMENT

None of the authors has any potential conflicts of interest to report.

## Supporting information


Figure S1.
Figure S2.

Table S1.

## Data Availability

The data that support the findings of this study are available in the supplementary material of this article.
