# Supplementary material for: Nanobubbles in vase water inhibit transpiration and prolong the vase life of cut chrysanthemum flowers
Source: Plant Environ Interact. 2023 Oct 24;4(6):309–16. doi: 10.1002/pei3.10124 (PMC10711642; doi:10.1002/pei3.10124)
Supplement: Supplementary file 1 — Figure S1. Figure S2. [file PEI3-4-309-s001.pptx]

## Slide 1
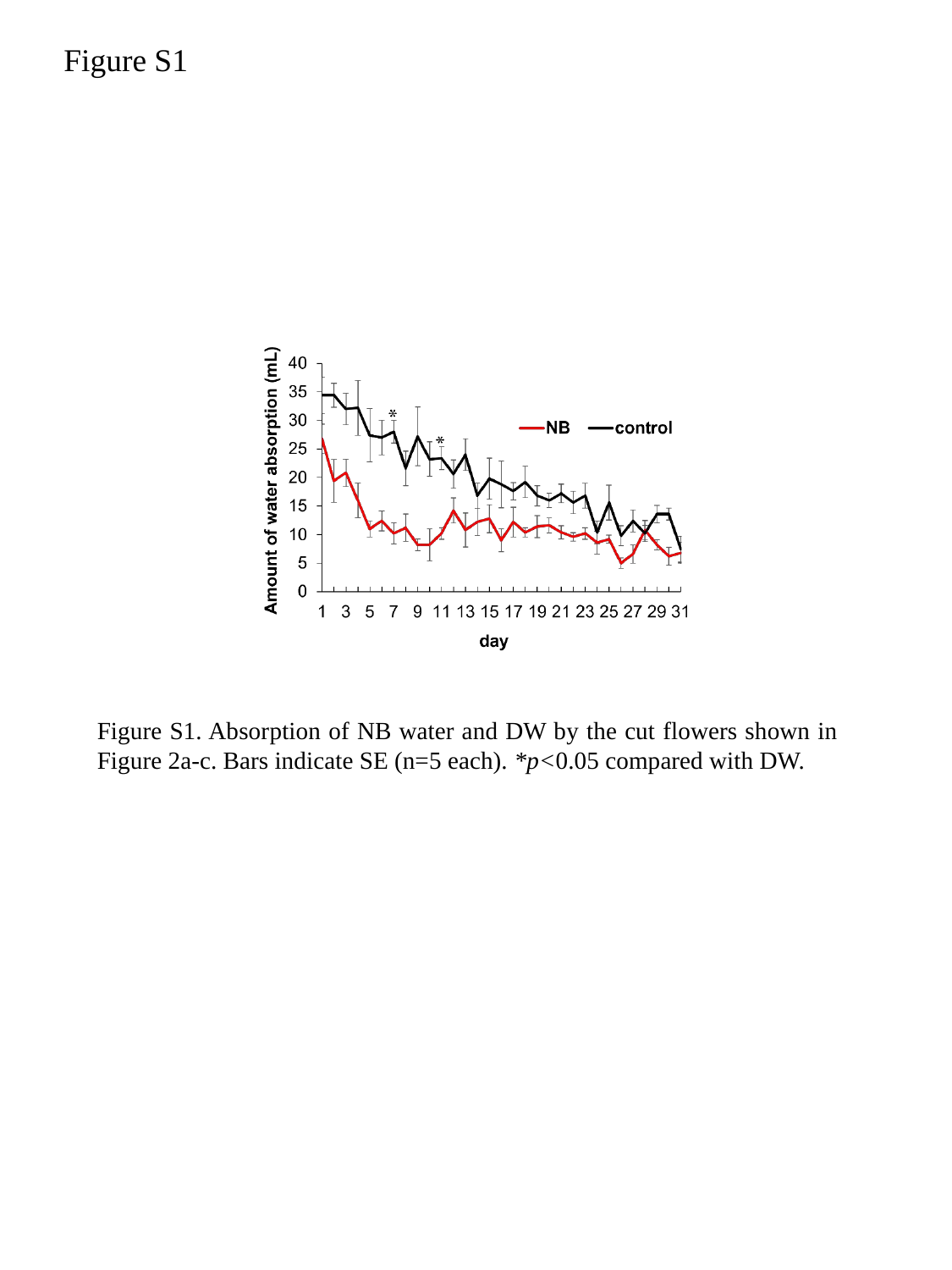

Figure S1
Figure S1. Absorption of NB water and DW by the cut flowers shown in Figure 2a-c. Bars indicate SE (n=5 each). *p<0.05 compared with DW.

## Slide 2
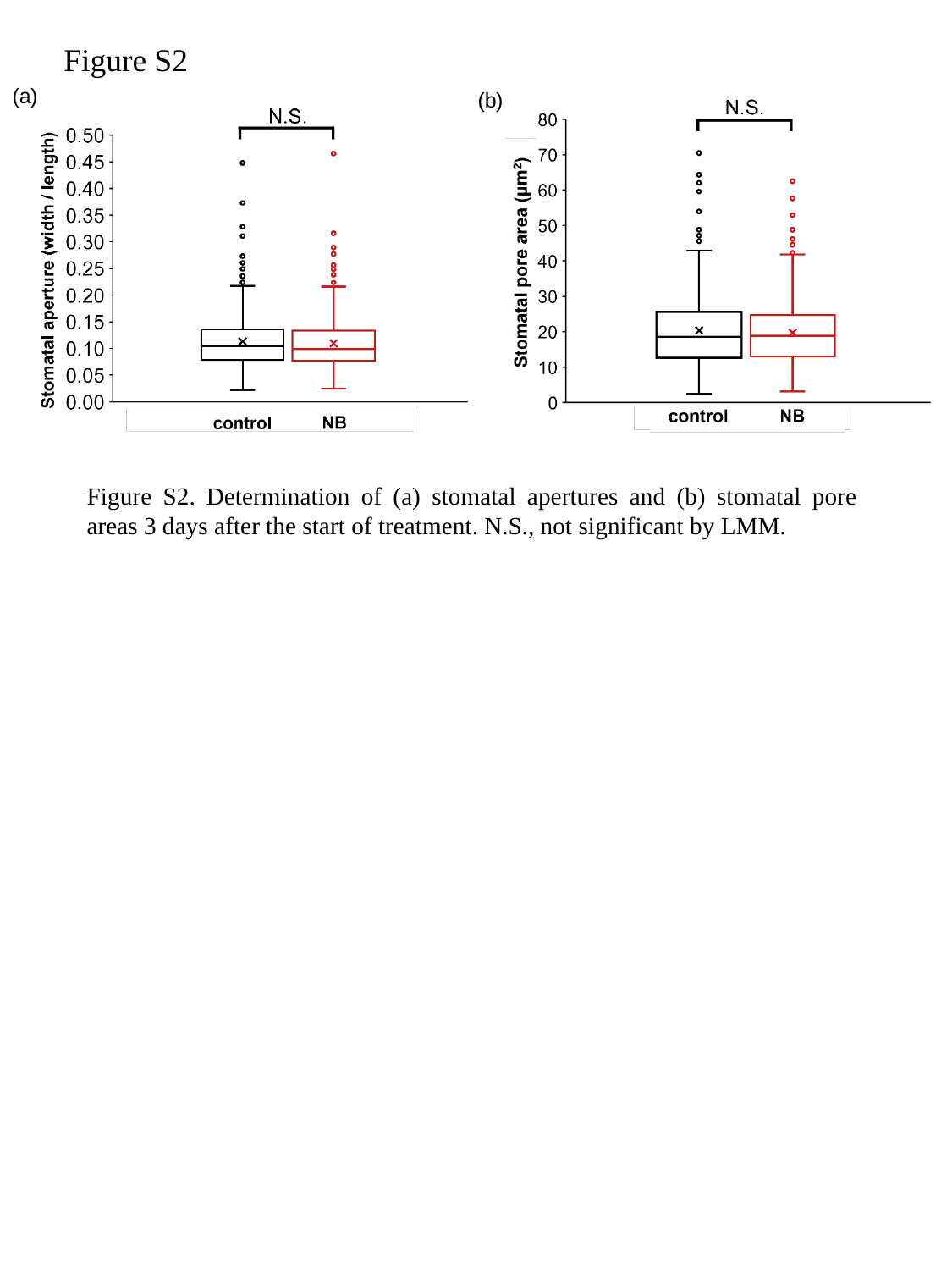

Figure S2
(a)
(b)
Figure S2. Determination of (a) stomatal apertures and (b) stomatal pore areas 3 days after the start of treatment. N.S., not significant by LMM.
