# Supplementary material for: Nanobubbles in vase water inhibit transpiration and prolong the vase life of cut chrysanthemum flowers
Source: Plant Environ Interact. 2023 Oct 24;4(6):309–16. doi: 10.1002/pei3.10124 (PMC10711642; doi:10.1002/pei3.10124)
Supplement: Supplementary file 2 — Table S1. [file PEI3-4-309-s002.docx]

Table S1. Evaluation of freshness of cut flowers based on morphological characteristics.

| Parameter | Criteria | Evaluation |
| --- | --- | --- |
| Flower opening | Start of opening of the petals of ray florets | A |
|  | Development of outer ray florets | B |
|  | Development of inner ray florets | C |
|  | Falling of petals | D |
| Browning of petals | No browning observed | A |
|  | Browning in part of the petals | C |
|  | Browning extending over entire petals | D |
| Curls of petals | Tension maintained in the petals (Check by touch) | A |
|  | Slightly soft petals (Check by touch) | B |
|  | Soft petals (Check by touch) | C |
|  | Shrinkage of petals (Check by observation) | D |
| Curls of leaves | Tension maintained in the leaves (Check by touch) | A |
|  | Slightly soft leaves (Check by touch) | B |
|  | Shrinkage of leaves, but recovery after cutting the lower part of the stems | C |
|  | Shrinkage of leaves are shrinking, but no recovery after cutting the lower part of the stems | D |
| Browning and etiolation of leaves | No browning or etiolation | A |
|  | Browning of lower leaves | B |
|  | Browning of middle and upper leaves | C |
|  | Browning and etiolation in more than half the leaves; with browning of all lower leaves. | D |
|  | Complete withering of lower leaves | E |

This table was based on the guidelines for freshness of chrysanthemum flowers published by the Japan Flower Promotion Center Foundation. Flowers with at least one rating of D or E or two or more C ratings were deemed to be at the end of their vase life.
